# Supplementary material for: Improving Access to Sexual Health Services in General Practice Using a Hub-and-Spoke Model: A Mixed-Methods Evaluation
Source: Int J Environ Res Public Health. 2022 Mar 25;19(7):3935. doi: 10.3390/ijerph19073935 (PMC8998128; doi:10.3390/ijerph19073935)
Supplement: Supplementary file 1 [file ijerph-19-03935-s001.zip › ijerph-1615312-supplementary.pdf]

Supplementary File S1: Features of the training for the spokes

Below are details of the assessment, support, training and education that Melbourne Sexual Health Centre provided to the three GP spokes to enable them to provide new STI services.

New GP clinic capabilities and services

Confidential appointment booking

Each practice chose a name for their sexual health appointments to make the booking process easier for them and the reception staff. This means that the patients don't have to discuss the reason for their visit until they speak with the clinician.

Model of Care

The nurse-initiated model, where the nurse completes the initial risk assessment, and then the patient sees the GP for clinical care, helps embed and strengthen the STI care patient pathway.

Bulk billing

The practices provide bulk billing appointments for STI testing, diagnosis and treatment, and some drop-in appointments for urgent cases.

HIV Prevention

They prescribe HIV post-exposure prophylaxis (PEP), stocked at their partner pharmacies.

They prescribe and manage patients requesting HIV pre-exposure prophylaxis (PrEP).

Injectable treatment for STIs stocked onsite

The clinics provide onsite injectable treatment of ceftriaxone and benzathine penicillin. This removes a known barrier to GP care of syphilis and gonorrhoea.

Connection to Stakeholder Groups

The practices have linked with the following agencies for support and education.

Thorn Harbour Health – This is a community-controlled organisation that develops and delivers effective community-driven health and well-being programs, advocating to reduce stigma and discrimination.

Family Planning Victoria. This organisation promotes reproductive and sexual health for all Victorians through their clinics, education and advocacy.

Rhed. This is a specialist service for the sex industry in Victoria which aims to improve the health and wellbeing of sex workers across Victoria.

The Victoria Non-Occupational Post Exposure Prophylaxis (NPEP) Service. They are responsible for the service administration, including the coordination of sites where NPEP is available throughout Victoria.

Training and education to GP clinic staff

Melbourne Sexual Health Centre assessed each of the clinics to identify their needs. Training and education were provided through face-to-face teaching in the GP clinics, teleconferencing, and visits to Melbourne Sexual Health Centre by GP staff.

Educational topics

Clinic processes
Introduction to STIs
Urethritis
Vaginal discharge
Sexual History Taking
Syphilis
Ano-rectal syndromes
Herpes Simplex and HPV

Specific skills

Sexual history taking for GPs: targeted to guide testing
STI screening including men who have sex with men and sex workers
Testing and management for chlamydia, gonorrhoea, Mycoplasma genitalium
HIV PrEP
Syphilis test results, antenatal screening and management
Vaccines
HPV
HIV testing and new diagnosis
HIV management
Adolescent health and STIs including schools

Nurse education package

STI overview including STI testing + case studies
Sexual history taking and role plays
Adolescent Sexual Health
Diversity in sexual health
Sex Work

Nurse Support Package
Nurse model for history taking and screening: item numbers
Nurses observation at Melbourne Sexual Health Centre
Melbourne Sexual Health Centre onsite visits to GP practices for set up and observation and support
Nurse sexual and reproductive health course at Melbourne Sexual Health Centre
Nurse support for school outreach
Nurse immuniser course paid for the practice nurses

Other education to support the clinics

Posters and flyers advertising sexual health service
Information regarding culturally appropriate care to vulnerable populations
Education session to the reception team on understanding vulnerable populations, including sex workers, the LGBTQ+ community
Administration team sit in at Melbourne Sexual Health Centre for observation.
Melbourne Sexual Health Centre pharmacy connection with GP partner pharmacies
Melbourne Sexual Health Centre counsellor connection with GP psychologists

Ongoing Support from Melbourne Sexual Health Centre to the GP Spokes

All clinical staff at the practices have access to the 1800 phone carried by a sexual health specialist at Melbourne Sexual Health Centre.
The GP clinics have a direct referral pathway to Melbourne Sexual Health Centre for clients with more complex STIs.
The GP psychologists and partner pharmacies have a direct connection to the Melbourne Sexual Health Centre counsellor and pharmacist for support and referral pathways.
The practice managers and Melbourne Sexual Health Centre nursing services manager have regular meetings for connection, troubleshooting and support.
The nurses and GPs have access and regular check-ins with the clinical staff for support and updates. They also ensure any new clinical staff have the education and sit in sessions.

Supplementary Table 1 summarises the impact of the GP hub and spoke model before and after implementation.

Supplementary Table S1

Before	After
Low self-rated knowledge of sexual health<br>Virtually all have not had any formal training in providing sexual health.	Upskilled GPs who feel more confident and competent to deliver sexual health care through their general practice/
Discomfort/awkwardness in initiating sexual history.	Feeling more confident in taking a comprehensive sexual history – having the sexual health clinic has given more time to do this, rather than squeezing this into an already busy GP consultation.<br>Having a consultation specifically for sexual health creates an environment where patients are more comfortable to open up.<br>Active integration of discussions about sexual health into the mental health plan and other health checks.
Had to refer cases of syphilis, HIV, PrEP, PEP to Melbourne Sexual Health Centre.	Able to handle more complex sexual health cases.
Uncommonly seeing priority populations, especially LGBTQ+ communities and female sex workers.<br>Concerns about conservative patient base – patients not comfortable in revealing sexuality identity.	Welcoming increased diversity of patients coming to the clinic.
Unclear which services could provide support or further management for complex cases.	Aware of where to find help.
Unaware of community-based organisations related to sexual health.	Aware of community resources – Resourcing Health & Education (RhED) for the sex industry, Thorne Harbour Health

Nurses not involved in sexual health – left it up to doctors.	Nurse upskilled and part of the team to provide sexual health care.
Local pharmacies are not stocked with HIV antiretrovirals.	Local pharmacies can now provide PEP and are listed on the getPEP website (https://www.getpep.info/).
General interest in learning more about sexual health.	Some GPs expressed interest in further upskilling, e.g. S100 prescriber course for HIV, Hepatitis.
Aware of needs for the surrounding community to have better sexual health services.	Able to provide this.
Unclear which STI tests to order for subpopulations.	Clarified system of appropriate testing for subpopulations – created swab collection guide.
Providing school program – but no routine offer of sexual health services to the school.	School program – planning to integrate sexual health discussions.
Cautious in not offending people.	Greater confidence in how to manage diverse clients professionally.
Difficulties in recruiting staff (in one general practice).	Recruited two new GPs interested in providing sexual health.

Supplementary File S2 Test Positivity for chlamydia, gonorrhoea, syphilis and HIV

Supplementary Figure S1 Chlamydia test positivity

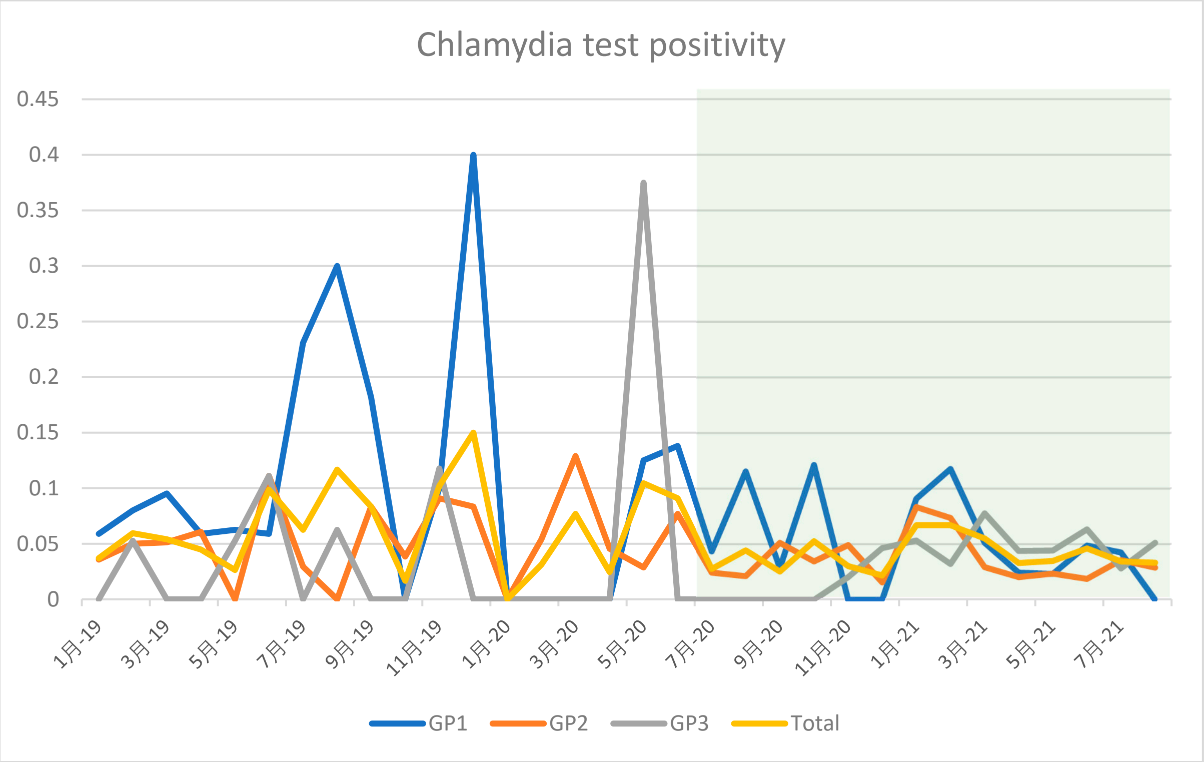

Green shaded area is the post-implementation period.

Supplementary Figure S2 Gonorrhoea test positivity

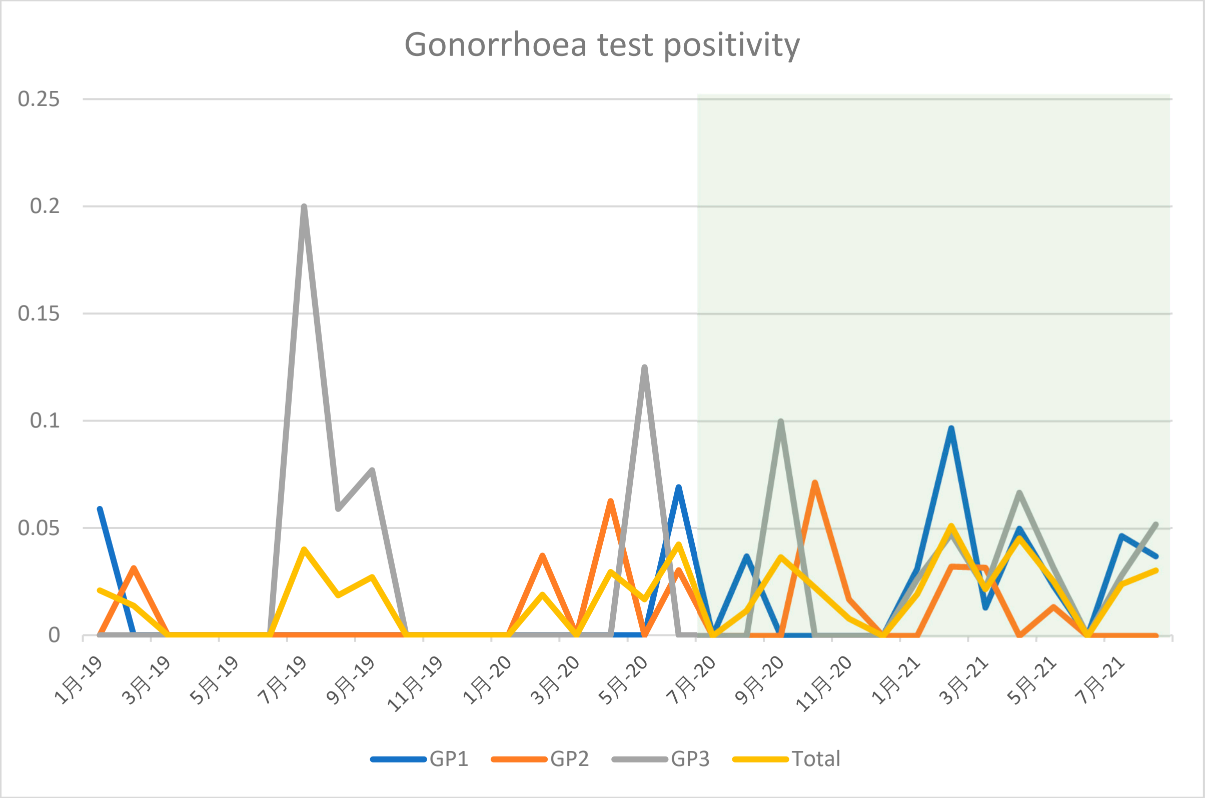

Green shaded area is the post-implementation period.

Supplementary Figure S3 Syphilis test positivity

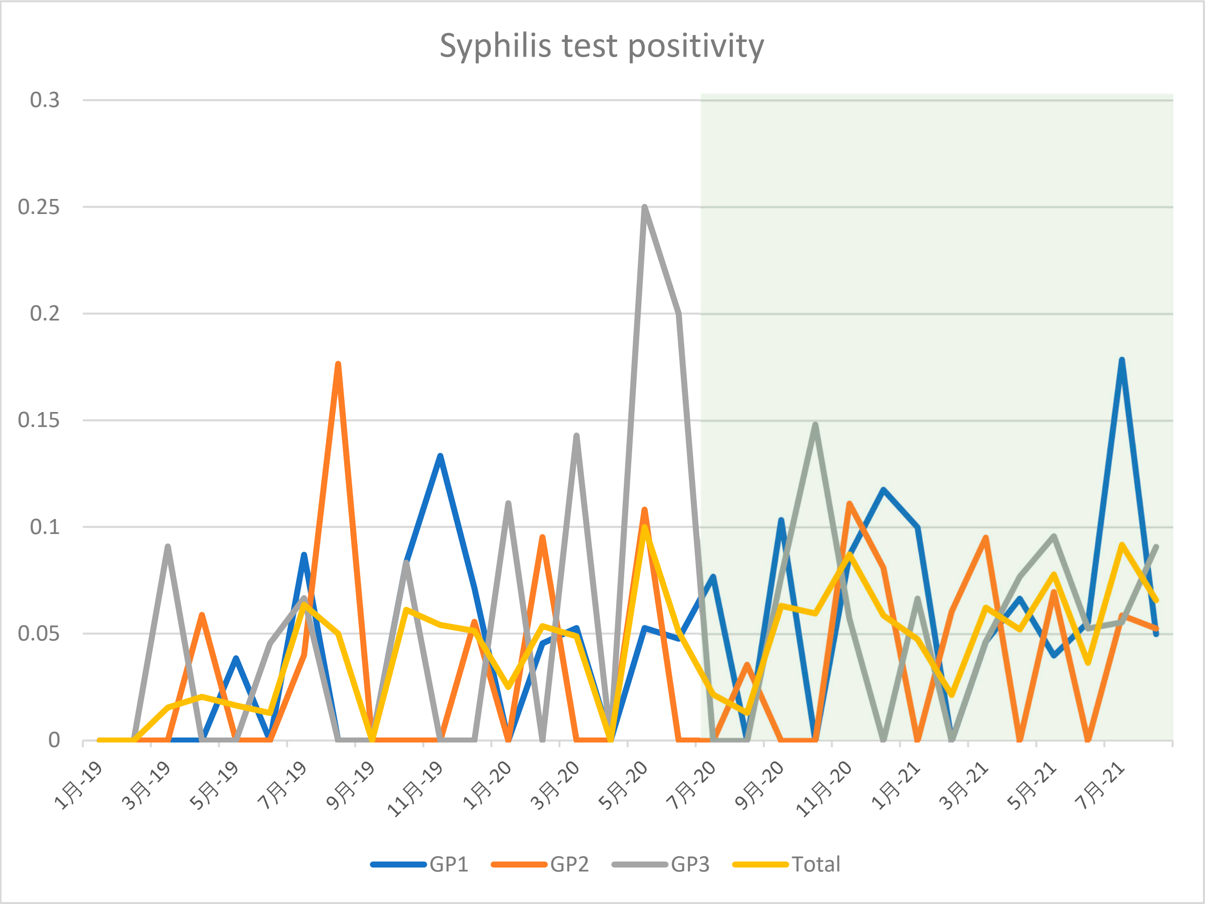

Green shaded area is the post-implementation period.

Supplementary Figure S4 HIV test positivity

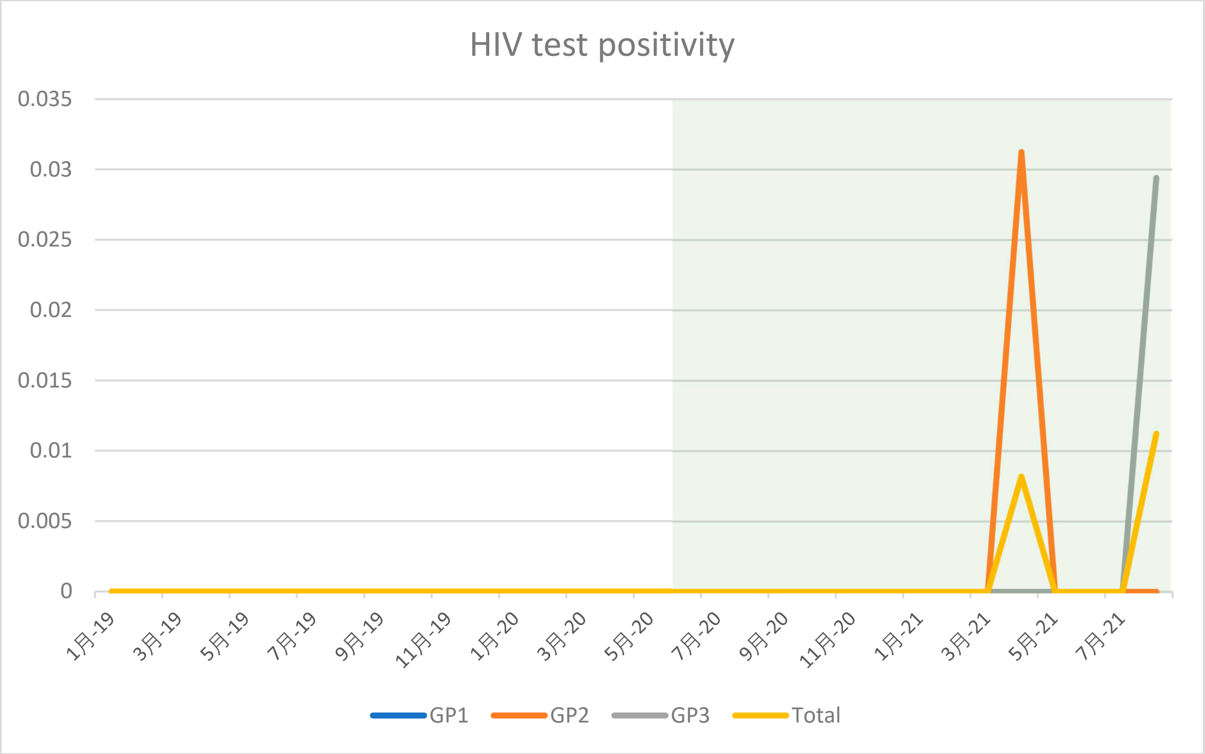

Green shaded area is the post-implementation period.

Supplementary File 3 Results from the qualitative interviews

This appendix captures the quotes from the qualitative interviews related to training needs, other challenges associated with delivering sexual health services, and areas for improvement for future Hub and Spoke models. Supplementary Table 2 provides the type of staff interviewed in the three general practices.

Supplementary Table S2. Type of staff interviewed

	Clinic 1	Clinic 2	Clinic 3
Doctors	2	6	9
Nurses	2	2	3
Administrative staff	1	5	3

Training needs

<u>General practitioners</u>

Discussions about sexual health.

'*taking a non-judgmental sexual history'.*

'*using culturally appropriate terms* [for LGBTQ+]'.

'*using the right pronouns… what words to use*'.

'*be more confident to ask questions* [related to sexual health]'.

'*I feel awkward bringing up the topic in non-sexual health-related consultations'.*

'how to disclose to partners about STIs … how to approach this'.

'how to promote sexual health opportunistically'.

Management of specific STI pathogens.

syphilis

gonorrhoea

Hepatitis B/C

herpes

anogenital warts

Mycoplasma genitalium

anogenital dermatology

Other management issues.

prescribing HIV pre-exposure prophylaxis (PrEP), post-exposure prophylaxis (PEP).

anoscopy/proctoscopy.

choosing the appropriate tests for different subpopulations.

how to take rectal swabs.

understanding and managing unique health challenges in LGBTQ+, esp transgender populations.

'*avoid perpetuating stigma and discrimination*'.

how to approach and manage sexual health for sex workers.

'*what are the legal requirements… tips for communication…. what they might find offensive*'.

'*using judgment-free language to avoid communication barriers*'.

understanding current HIV/STI guidelines and how to access them.

contact tracing.

Nurses

Discussions about sexual health.

'how to start a discussion about sexual health without the person feeling awkward'.

'how to deal with people – make them feel more comfortable in discussing about sexual health'.

'what language to use when speaking to sexual minorities, sex workers, young people'.

'*make as inviting and approachable a subject for clinic*'.

desired '*comprehensive understanding of STIs*'.

Resources.

list of online resources related to sexual health.

where to get patient education resources.

How to 'work as a team with the doctor' in this new service.

Administrative staff

Interested in providing 'holistic care in GP including vulnerable, underprivileged'.

'*more tools/training to deal with them*'.

'*not to make patients feel awkward… use the right terminology'.*

'*to be more sensitive, tactful, discreet*'.

Resources.

Opportunities to learn from other clinics or videos to demonstrate how administrative staff deal with patients is managed by other administrative staff.

Manual for stocks – which medications, swabs are needed.

Advertisement/promotion of sexual health services when available.

Other challenges of providing sexual health services

General practitioners

Promotion of the sexual health service to patients and the community.

'Clinic not known in the community to provide sexual health, so sex workers not attending for certificates'.

Patients not comfortable in '*coming out*'.

Reactions from other patients.

'*GP practice serves a multicultural area with many patients coming from 'conservative' cultures*'.

'*I am seeing mostly Asian patients who may not be comfortable to just come in for sexual health*'.

Understanding the logistics and role of doctors/nurses and how the new sexual service will look.

'*workflow in the clinic*'.

Nurses

To be actively involved in taking a sexual health history.

Improving reminder systems to recall patients.

No system was set up for contact tracing or recalling patients for a test of reinfection.

Coordinating 'limited nurse time'.

How to integrate sexual health into school programs (high school).

Administrative staff

Clinic flow and turnover of staff.

More promotion about sexual health.

Areas for improvement

Training/Preparation.

To provide more flexible timing for the lectures with a preference for face-to-face rather than online teleconferencing.

Some staff felt the pressure to be ready on time.

'*a bit intimidating initially but ended up being not too bad*'.

'*was stressful but could see the benefits*'.

Promotion of the service.

All three clinics expressed the desire to see more patients and were eager for a greater diversity of patients (including sex workers, LGBTQ+ communities).

They felt that the sexual health clinic concept may still be foreign to their patients and the surrounding community. They suggested more advertising through the Primary Health Networks, Shopping Centres, and access to posters/videos they could put in their waiting rooms.

Providing clear distinction of "hub" and "spoke" services to patients.

Providing sexual health services in a GP clinic meant that some patients may perceive the service to be less anonymous (compared to attending Melbourne Sexual Health Centre).

There were also comments regarding managing patients' expectations: that they were upskilled GPs, not specialists in sexual health.

Out of pocket costs for non-Medicare holders.

Clinics are bulk-billing clinics, but the cost may be an issue for non-Medicare cardholders.

Quality control.

Some were interested in opportunities for routine refresher training (e.g. once a year).

Some expressed the value of regularly sitting down with all staff (including a representative from Melbourne Sexual Health Centre) to reflect on experiences and learn from other general practices in other 'spokes'.

The need to provide training for new staff joining the GP.

Before training

Pre-training, the qualitative interviews uncovered various gaps in sexual health knowledge and practices. Most GPs referred cases related to HIV pre-exposure prophylaxis (PrEP), HIV post-exposure prophylaxis (PEP) and syphilis to Melbourne Sexual Health Centre. All staff welcomed the opportunity to update their sexual health knowledge and provide sexual health services to their GP clientele.

Most doctors reported not receiving any extra training in sexual health ever, but there was at least one GP in each clinic with some experience or training. This included attending webinars on HIV pre-exposure prophylaxis (PrEP), previous jobs related to obstetrics and gynaecology, attendance of lectures hosted by local sexual health

services. All staff interviewed expressed enthusiasm regarding the opportunity to upskill in sexual health through this collaboration. None expressed any concerns with managing members of priority populations (LGBTQ+, sex workers, people who inject drugs or Aboriginal and Torres-Strait Islanders, people in correctional facilities, refugees). They would '*treat all patients the same… with respect*', but the majority expressed they would appreciate additional training to '*deal with these people… better understand them*' and '*how to deal with patients – discreet, not make them feel awkward*'. Aside from young people, most GPs reported rarely providing consultations with members of priority populations.

GPs expressed confidence in conducting genital exams but were less confident in managing anogenital dermatological pathology. When a complex sexual health case (e.g. syphilis or HIV) was encountered, most GPs would usually refer to or speak with a sexual health or infectious disease physician. GPs reported difficulties in bringing up sexual health within the majority of GP consults and would only explicitly ask about sexual health opportunistically during a consultation related to the cervical screening test, contraception or pregnancy to discuss about sexual health. There were uncertainties about how best to normalise sexual health discussions with male patients.

All nurses from the three GP practices had no experience routinely taking a comprehensive sexual history. The majority of nurses felt confident and could administer injections (including benzathine penicillin), vaccinations, and take blood for pathology. Some nurses had undergone cervical screening test training so were comfortable doing female genital examinations, including speculum examination.

Post-training interviews

All participants reported increases in *knowledge* level and *confidence* in offering STI testing and managing more variety of sexual health cases (including syphilis, PrEP, PEP). They expressed they were able to find the information they required quickly. GPs also believed they were now providing a better service to their patients: '*I am able to take a more comprehensive sexual history with dedicated time rather than squeezing this into a busy GP consultation*'. The majority of GPs also mentioned they were more confident in discussing sexual health '*in a way that is not awkward*'. One GP said that patients were watching him closely and were initially guarded but would relax when he could demonstrate his knowledge in this field. GPs felt confident they had the necessary backup (Melbourne Sexual Health Centre hotline, lecture materials, websites) for more complex cases.

Some doctors reported an increased interest in sexual health, and the majority appreciated the importance of sexual health and started integrating this into other parts of their clinical practices. They expressed greater awareness of other local community organisations previously unknown to them (e.g. for sex workers or LGBTQ+ people). One clinic had a key staff leave during the pilot phase. There were difficulties in recruiting a replacement; however, due to assistance from Melbourne Sexual Health Centre, they could recruit two new GPs who had a special interest in sexual health, to join their general practice.
